# Pharmacokinetics, safety, and tolerability of HRS-8427, a novel anti-carbapenem-resistant cephalosporin, in healthy subjects

**DOI:** 10.1128/aac.00694-25

**Published:** 2026-03-03

**Authors:** Tengrui Yin, Biao Qu, Qian Zhang, Sheng Xu, Yijue Wu, Hao Jiang, Yuanyuan Huang, Wei Hu

**Affiliations:** 1 Jiangsu Hengrui Medicine Co. Ltd. 425579 , Lianyungang, People's Republic of China; 2 Department of Clinical Pharmacology, The Second Affiliated Hospital of Anhui Medical University 687310 , Hefei, People's Republic of China; Providence Portland Medical Center, Portland, Oregon, USA

**Keywords:** HRS-8427, pharmacokinetics, cephalosporin, gram-negative bacteria, siderophores

## Abstract

HRS-8427 is a novel parenteral siderophore cephalosporin that shows potent efficacy against various gram-negative bacteria, including carbapenem-resistant strains, *in vitro* and in preclinical models of infection. It underwent a single-dose ascending study (400–6,000 mg) and a multiple-dose ascending study (1,000–3,000 mg q8h) in healthy Chinese adults to evaluate pharmacokinetics (PK), safety, and tolerability. A total of 62 treatment-emergent adverse events (TEAEs) occurred in 29 subjects during the treatment period, resulting in a TEAE incidence of 36.7% (29/79). There were no serious adverse events (AEs) observed in either study. Three subjects (one in the 2,000 mg single-dose group and two in the 3,000 mg multi-dose group) withdrew from the trial due to AEs. Approximately dose-proportional increases in the maximum plasma concentration (*C*_max_) and the area under the concentration-time curve (AUC) were observed across the single-dose range of 400–6,000 mg, although the drug exposure increased slightly less than the dose. The mean plasma half-life of HRS-8427 was 3.89 to 4.28 h. HRS-8427 was primarily excreted unchanged in the urine (62.13% to 71.12% of the dose). There was minimal systemic accumulation of *C*_max_ and AUC by dosing q8h, and the PK of HRS-8427 did not change with multiple dosing. This study indicates that both single and multiple intravenous doses of HRS-8427, up to 6,000 mg and 2,000 mg, respectively, are well tolerated in healthy subjects and show generally linear pharmacokinetics at doses up to 6,000 mg.

This study is registered with the Chinese Clinical Trial registry as ChiCTR2200062570, and ClinicalTrials.gov as NCT07070375, NCT06144060, NCT06569056, NCT06841731, NCT07049562, NCT07049107, and NCT07073157.

## INTRODUCTION

The rising incidence of infections caused by drug-resistant bacterial pathogens is a critical global health crisis, responsible for approximately 700,000 deaths each year. This alarming trend is projected to lead to as many as 10 million deaths annually by 2050 (1, 2). It is noteworthy that β-lactam antibiotics have been among the most effective and widely used antibacterial agents, but the emergence of resistance to these drugs has become a major antimicrobial resistance, highlighting the urgent need for effective solutions.

Of particular concern is the rapid emergence of serious nosocomial infections caused by gram-negative pathogens, such as *Pseudomonas aeruginosa* and *Acinetobacter baumannii*, as well as carbapenem-resistant *Enterobacterales*, which render the most commonly used class of antibiotics, β-lactams, ineffective. Thus, there is an urgent need to develop new antibiotics to combat the increasing number of multidrug-resistant gram-negative bacterial strains. The mechanisms responsible for carbapenem resistance in gram-negative bacteria include the following: (i) decreased permeability, related to the inability of antibiotics to reach their sites of action by reducing the entry into their outer cell walls through the porin channels. (ii) Overexpression of the efflux pump, removing the antibiotic from its site of action before it can act. (iii) Mutation and transformation in antibiotic target structures, decreasing affinity for the antibiotics, which do not work completely and behave with reduced efficiency. (iv) Modification of antibiotics by the hydrolysis of the molecule (3, 4).

Here, we report our successful efforts to manifest siderophore antibiotics HRS-8427, which modifies the chain of the cephalosporin with special chemical group to chelate iron. This optimized combination allows HRS-8427 to be transported into bacterial cells through the bacterial iron transport system, actively. Non-clinical studies have demonstrated that HRS-8427 exhibits potent antibacterial activity against aerobic gram-negative pathogens, including *Enterobacteriaceae*, *P. aeruginosa*, and *A. baumannii*. In a murine urinary tract infection model, HRS-8427 significantly reduced bacterial burdens in both the bladder and kidneys. Furthermore, preclinical findings indicated that the bactericidal activity of HRS-8427 increased with dosing frequency, suggesting a time-dependent killing profile. (All data referenced are unpublished.) The only siderophore antibiotics that have been approved for clinical use are cefiderocol (5, 6). Our successful modification of HRS-8427 may bring more choices for resistance to β-lactam antibiotics caused by gram-negative pathogens.

To date, HRS-8427 has completed a first-in-human (FIH) study (ChiCTR2200062570), a PK study in subjects with renal impairment (NCT07070375), a PK study evaluating the intrapulmonary penetration (ChiCTR2300072350), and a Phase II study in patients with complicated urinary tract infections, including acute pyelonephritis (cUTI/AP) (NCT06144060). A Phase III clinical trial in patients with complicated urinary tract infections (NCT06569056), a Phase II study in patients with bacterial pneumonia (NCT06841731), a mass-balance study (NCT07049562), and two Phase I studies evaluating drug-drug interaction (NCT07049107 and NCT07073157) are currently ongoing. All data are currently unpublished.

The primary aim of this FIH study was to evaluate the safety, tolerability, and pharmacokinetics of HRS-8427 in single and multiple doses in healthy Chinese participants.

## RESULTS

### Subjects

In this study, a total of 81 subjects were enrolled and randomized, with two subjects discontinuing the trial before receiving medication and 79 subjects receiving medication, with 50 subjects in a single-dose study (SAD) and 29 subjects in a multi-dose study (MAD). Thus, of all the 81 subjects included in the full analysis set (FAS) (51 subjects in single-dose study, 30 subjects in multi-dose study) and 79 subjects included in the safety analysis set (SS). Additionally, as Table 1 shows, with the exception of the 16-placebo group subjects, 63 subjects were included in the pharmacokinetic concentration set (PKCS) (40 in SAD and 23 in MAD). During the course of the study, three subjects (one in 2,000 mg single-dose group and two in 3,000 mg multi-dose group) did not complete the HRS-8427 treatment and withdrew from the trial due to AE hypersensitivity. As pharmacokinetic parameter data were not available for those subjects, 60 subjects were included in the pharmacokinetic parameter set (PKPS) (39 in single-dose study and 21 in multi-dose study). Table 2 shows the demographics and baseline characteristics of all dosed subjects.

**TABLE 1:** The division of the subject dataset

	SAD 400 mg n (%)	SAD 1,000 mg n (%)	SAD 2,000 mg n (%)	SAD 4,000 mg n (%)	SAD 6,000 mg n (%)
Randomized subjects	8	8	8	9	8
Full analysis set	8 (100.0)	8 (100.0)	8 (100.0)	9 (100.0)	8 (100.0)
Safety analysis set	8 (100.0)	8 (100.0)	8 (100.0)	8 (88.9)^*a*^	8 (100.0)
PK concentration set	8 (100.0)	8 (100.0)	8 (100.0)	8 (88.9)^*a*^	8 (100.0)
PK parameter set	8 (100.0)	8 (100.0)	7 (87.5)^*b*^	8 (88.9)^*a*^	8 (100.0)

	MAD 1,000 mg n (%)	MAD 2,000 mg n (%)	MAD 3,000 mg n (%)	Placebo n (%)	Total n (%)
Randomized subjects	8	8	8	16	81
Full analysis set	8 (100.0)	8 (100.0)	8 (100.0)	16 (100.0)	81 (100.0)
Safety analysis set	7 (87.5)^*a*^	8 (100.0)	8 (100.0)	16 (100.0)	79 (97.5)
PK concentration set	7 (87.5)^*a*^	8 (100.0)	8 (100.0)	0 (0.0)	63 (77.8)
PK parameter set	7 (87.5)^*a*^	8 (100.0)	6 (75.0)^*c*^	0 (0.0)	60 (74.1)

^*a*^ One subject withdrew from the study before administration.

^*b*^ One subject withdrew from the study due to hypersensitivity.

^*c*^ Two subjects withdrew from the study due to hypersensitivity.

**TABLE 2:** Demographics and baseline characteristics^*a*^

	Single-dose study						Multi-dose study			
	400 mg (*N* = 8)	1,000 mg (*N* = 8）	2,000 mg (*N* = 8)	4,000 mg (*N* = 9)	6,000 mg (*N* = 8)	Placebo (*N* = 10)	1,000 mg (n = 8)	2,000 mg (n = 8)	3,000 mg (n = 8)	Placebo (n = 6)
Age (years)										
Mean (SD)	28.5 (7.19)	26.8 (4.86)	27.9 (7.83)	29.2 (5.76)	28.9 (5.59)	27.9 (5.63)	29.8 (8.08)	29.9 (6.17)	30.9 (4.94)	29.5 (6.53)
Min, Max	20, 40	19, 34	19, 44	21, 36	20, 35	20, 38	21, 42	21, 38	23, 37	20, 36
Gender, n(%)										
male	4 (50.0)	4 (50.0)	4 (50.0)	4 (44.4)	4 (50.0)	5 (50.0)	4 (50.0)	4 (50.0)	4 (50.0)	3 (50.0)
female	4 (50.0)	4 (50.0)	4 (50.0)	5 (55.6)	4 (50.0)	5 (50.0)	4 (50.0)	4 (50.0)	4 (50.0)	3 (50.0)
Race, n(%)										
Asian	8 (100.0)	8 (100.0)	7 (87.5)	9 (100.0)	7 (87.5)	10 (100.0)	8 (100.0)	7 (87.5)	7 (87.5)	6 (100.0)
Non-Asian	0 (0.0)	0 (0.0)	1 (12.5)	0 (0.0)	1 (12.5)	0 (0.0)	0 (0.0)	1 (12.5)	1 (12.5)	0 (0.0)
Height (cm)										
Mean (SD)	165.56 (9.225)	165.69 (10.021)	168.56 (11.675)	164.06 (9.551)	169.63 (9.924)	167.65 (6.617)	165.38 (7.029)	163.94 (6.951)	166.25 (8.924)	164.17 (8.424)
Min, Max	152.5, 175.5	151.5, 178.0	155.5, 190.5	148.0, 184.0	153.5, 184.5	157.0, 179.5	151.5, 172.0	150.5, 172.0	156.5, 183.5	155.0, 173.5
Weight (kg)										
Mean (SD)	62.91 (8.101)	62.71 (10.695)	65.20 (11.935)	57.97 (7.775)	64.61 (8.595)	63.27 (5.217)	60.46 (8.039)	57.25 (5.487)	60.68 (8.161)	57.48 (7.143)
Min, Max	49.6, 73.7	48.0, 76.1	53.2, 84.8	49.1, 73.7	53.3, 75.2	56.7, 72.1	46.8, 68.8	50.1, 66.8	49.5, 76.0	49.3, 68.7
BMI (kg/m^2^)										
Mean (SD)	22.86 (1.143)	22.68 (1.801)	22.78 (1.781)	21.51 (1.630)	22.41 (1.645)	22.53 (1.615)	22.04 (1.661)	21.33 (1.921)	21.93 (1.796)	21.28 (1.385)
Min, Max	21.2, 24.6	20.8, 25.0	19.3, 25.0	19.3, 24.4	19.8, 24.7	19.4, 24.7	20.0, 24.0	19.1, 24.1	19.3, 25.2	19.5, 23.5
eGFR (mL/min/1.73 m^2^)										
Mean (SD)	132.60(19.34)	148.50(24.36)	150.30(32.15)	139.60(26.45)	144.90(21.00)	147.30(28.39)	138.20(21.53)	121.60(17.31)	140.90(21.78)	118.60(20.53)
Min, Max	98.30,157.40	127.40,203.50	108.40,208.30	94.40,189.10	115.30,175.20	111.30,185.10	112.20,166.00	102.60,156.20	102.30,167.90	84.10,144.50

^*a*^ SD, standard deviation; BMI, body mass index; kg, kilogram; N, number of subjects in the full analysis set by study arm; n, number of subjects in the specified category; %, n/N*100.

### Safety

The safety and tolerability of HRS-8427 were generally well tolerated in the single-dose and multiple-dose studies (Tables 3 and 4). In this study, a total of 62 treatment-emergent adverse events (TEAEs) occurred in 29 subjects during the treatment period, resulting in a TEAE incidence of 36.7% (29/79). Three subjects withdrew from the study due to hypersensitivity reactions (one subject in SAD and two subjects in MAD) after treatment.

**TABLE 3:** Incidence of adverse events in the single-dose study^*a*^ (safety analysis set)

System organ class (SOC) Preferred term (PT)	400 mg (*N* = 8)		1,000 mg (*N* = 8)		2,000 mg (*N* = 8)		4,000 mg (*N* = 8)		6,000 mg (*N* = 8)		Total (*N* = 40)		Placebo (*N* = 10)	
	E	n (%)	E	n (%)	E	n (%)	E	n (%)	E	n (%)	E	n (%)	E	n (%)
No. of subjects with TEAE	1	1 (12.5)	2	2 (25.0)	2	2 (25.0)	3	2 (25.0)	4	3 (37.5)	12	10 (25.0)	1	1 (10.0)
Immune system disorders	0	0 (0.0)	0	0 (0.0)	1	1 (12.5)	0	0 (0.0)	0	0 (0.0)	1	1 (2.5)	0	0 (0.0)
Hypersensitivity	0	0 (0.0)	0	0 (0.0)	1	1 (12.5)	0	0 (0.0)	0	0 (0.0)	1	1 (2.5)	0	0 (0.0)
Investigations	1	1 (12.5)	1	1 (12.5)	0	0 (0.0)	2	2 (25.0)	2	2 (25.0)	6	6 (15.0)	1	1 (10.0)
Protein urine present	0	0 (0.0)	1	1 (12.5)	0	0 (0.0)	0	0 (0.0)	1	1 (12.5)	2	2 (5.0)	1	1 (10.0)
Red blood cells urine positive	0	0 (0.0)	0	0 (0.0)	0	0 (0.0)	1	1 (12.5)	0	0 (0.0)	1	1 (2.5)	0	0 (0.0)
Electrocardiogram ST segment elevation	0	0 (0.0)	0	0 (0.0)	0	0 (0.0)	0	0 (0.0)	1	1 (12.5)	1	1 (2.5)	0	0 (0.0)
Lipase increased	0	0 (0.0)	0	0 (0.0)	0	0 (0.0)	1	1 (12.5)	0	0 (0.0)	1	1 (2.5)	0	0 (0.0)
Blood cholesterol increased	1	1 (12.5)	0	0 (0.0)	0	0 (0.0)	0	0 (0.0)	0	0 (0.0)	1	1 (2.5)	0	0 (0.0)
Infections and infestations	0	0 (0.0)	1	1 (12.5)	0	0 (0.0)	0	0 (0.0)	0	0 (0.0)	1	1 (2.5)	0	0 (0.0)
Gastroenteritis	0	0 (0.0)	1	1 (12.5)	0	0 (0.0)	0	0 (0.0)	0	0 (0.0)	1	1 (2.5)	0	0 (0.0)
Gastrointestinal disorders	0	0 (0.0)	0	0 (0.0)	1	1 (12.5)	1	1 (12.5)	2	2 (25.0)	4	4 (10.0)	0	0 (0.0)
Nausea	0	0 (0.0)	0	0 (0.0)	0	0 (0.0)	0	0 (0.0)	1	1 (12.5)	1	1 (2.5)	0	0 (0.0)
Mouth ulceration	0	0 (0.0)	0	0 (0.0)	0	0 (0.0)	0	0 (0.0)	1	1 (12.5)	1	1 (2.5)	0	0 (0.0)
Diarrhea	0	0 (0.0)	0	0 (0.0)	0	0 (0.0)	1	1 (12.5)	0	0 (0.0)	1	1 (2.5)	0	0 (0.0)
Abdominal pain	0	0 (0.0)	0	0 (0.0)	1	1 (12.5)	0	0 (0.0)	0	0 (0.0)	1	1 (2.5)	0	0 (0.0)

^*a*^ The data are shown as the number of subjects (number of events), percentage of subjects with adverse events. Denominators for the percentages are the number of subjects in the safety population in each treatment group.

**TABLE 4:** Incidence of adverse events in the multi-dose study^*a*^

System organ class (SOC) Preferred term (PT)	1,000 mg (*N* = 7)		2,000 mg (*N* = 8)		3,000 mg (*N* = 8)		Total (*N* = 23)		Placebo (*N* = 6)	
	E	n (%)	E	n (%)	E	n (%)	E	n (%)	E	n (%)
No. of subjects with TEAE	6	4 (57.1)	8	5 (62.5)	31	8 (100.0)	45	17 (73.9)	4	1 (16.7)
Immune system disorders	0	0 (0.0)	0	0 (0.0)	2	2 (25.0)	2	2 (8.7)	0	0 (0.0)
Hypersensitivity general disorders and administration	0	0 (0.0)	0	0 (0.0)	2	2 (25.0)	2	2 (8.7)	0	0 (0.0)
site conditions	0	0 (0.0)	2	2 (25.0)	3	3 (37.5)	5	5 (21.7)	0	0 (0.0)
Puncture site pain	0	0 (0.0)	2	2 (25.0)	2	2 (25.0)	4	4 (17.4)	0	0 (0.0)
Chest discomfort	0	0 (0.0)	0	0 (0.0)	1	1 (12.5)	1	1 (4.3)	0	0 (0.0)
Investigations	5	4 (57.1)	6	5 (62.5)	8	5 (62.5)	19	14 (60.9)	1	1 (16.7)
Protein urine present	1	1 (14.3)	3	3 (37.5)	7	5 (62.5)	11	9 (39.1)	0	0 (0.0)
Urinary occult blood positive	0	0 (0.0)	1	1 (12.5)	1	1 (12.5)	2	2 (8.7)	0	0 (0.0)
White blood cell counts increased	0	0 (0.0)	1	1 (12.5)	0	0 (0.0)	1	1 (4.3)	0	0 (0.0)
Lipase increased	0	0 (0.0)	0	0 (0.0)	0	0 (0.0)	0	0 (0.0)	0	0 (0.0)
Blood pressure decreased	2	1 (14.3)	0	0 (0.0)	0	0 (0.0)	2	1 (4.3)	1	1 (16.7)
Blood fibrinogen decreased	1	1 (14.3)	0	0 (0.0)	0	0 (0.0)	1	1 (4.3)	0	0 (0.0)
Blood cholesterol increased	1	1 (14.3)	1	1 (12.5)	0	0 (0.0)	2	2 (8.7)	0	0 (0.0)
Nervous system disorders	0	0 (0.0)	0	0 (0.0)	5	3 (37.5)	5	3 (13.0)	1	1 (16.7)
Headache	0	0 (0.0)	0	0 (0.0)	5	3 (37.5)	5	3 (13.0)	1	1 (16.7)
Infections and infestations	1	1 (14.3)	0	0 (0.0)	1	1 (12.5)	2	2 (8.7)	0	0 (0.0)
Upper respiratory tract infection	0	0 (0.0)	0	0 (0.0)	1	1 (12.5)	1	1 (4.3)	0	0 (0.0)
Pulpitis dental	1	1 (14.3)	0	0 (0.0)	0	0 (0.0)	1	1 (4.3)	0	0 (0.0)
Gastrointestinal disorders	0	0 (0.0)	0	0 (0.0)	12	3 (37.5)	12	3 (13.0)	2	1 (16.7)
Nausea	0	0 (0.0)	0	0 (0.0)	7	2 (25.0)	7	2 (8.7)	0	0 (0.0)
Mouth ulceration	0	0 (0.0)	0	0 (0.0)	1	1 (12.5)	1	1 (4.3)	0	0 (0.0)
Diarrhea	0	0 (0.0)	0	0 (0.0)	1	1 (12.5)	1	1 (4.3)	0	0 (0.0)
Abdominal pain	0	0 (0.0)	0	0 (0.0)	1	1 (12.5)	1	1 (4.3)	0	0 (0.0)
Abdominal distension	0	0 (0.0)	0	0 (0.0)	2	1 (12.5)	2	1 (4.3)	0	0 (0.0)
Vomiting	0	0 (0.0)	0	0 (0.0)	0	0 (0.0)	0	0 (0.0)	2	1 (16.7)

^*a*^ The data are shown as the number of subjects (number of events) and percentage of subjects with adverse events. Denominators for the percentages are the number of subjects in the safety population in each treatment group.

In the single-dose study, a total of 13 TEAEs were reported by 11 subjects, with 10 receiving HRS-8427 (10/40, 25%) and one receiving placebo (1/10, 10%). The incidence of TEAEs in the 400, 1,000, 2,000, 4,000, and 6,000 mg group was 12.5%, 25%, 25%, 25%, and 37.5% respectively, and was not dose-related. All TEAEs were of mild intensity. Nine TEAEs reported by 8 subjects in the HRS-8427 groups were considered to be related to the study drug: protein urine present (two events in two subjects), and one event each of hypersensitivity, red blood cells urine-positive, electrocardiogram ST-segment elevation, nausea, mouth ulceration, diarrhea, and abdominal pain. In the placebo group, one TEAE occurred and was considered not related to study treatment. The incidence and severity of all TEAEs showed no apparent dose dependency.

In the multiple-dose study, a total of 49 TEAEs were reported by 18 subjects, with 17 receiving HRS-8427 (17/23, 73.9%) and one receiving placebo (1/6, 16.7%). The incidence of TEAEs in the 1,000, 2,000, and 3,000 mg groups was 57.1%, 62.5%, and 100%, respectively and showed a slight dose dependence. All the TEAEs were mild intensity, except for one TEAE that was moderate (upper respiratory tract infection in the 3,000 mg group). Thirty-four TEAEs that occurred in 14 subjects (14/23, 60.9%) were considered possibly or probably related to the study treatment: hypersensitivity (two events in two subjects, 2/23, 8.7%), protein urine present (11 events in nine subjects, 9/23, 39.1%), urinary occult blood positive (two events in two subjects, 2/23, 8.7%), headache (five events in three subjects, 3/23, 13.0%), nausea (seven events in two subjects, 2/23, 8.7%), abdominal distension (two events in one subject, 1/23, 4.3%) and one event each of a pulpitis dental, diarrhea, abdominal pain, and decreased blood fibrinogen levels. In the placebo group, four TEAEs occurred in one subject, and all were considered not related to study treatment.

The incidence of protein urine present may demonstrate dose dependence with one event (1/7, 14.3%) in the 1,000 mg group, three events in three subjects (3/8, 37.5%) in the 2,000 mg group, and seven events in five subjects (5/8, 62.5%) in the 3,000 mg group, respectively. However, all of the above TEAEs were mild in severity and recovered without treatment, which indicated controlled safety risk.

All the TEAEs resolved spontaneously without treatment. No deaths or other serious adverse events (SAEs) were noted. No concomitant medication or treatment was used.

### Pharmacokinetics in plasma

**Fig 1:**
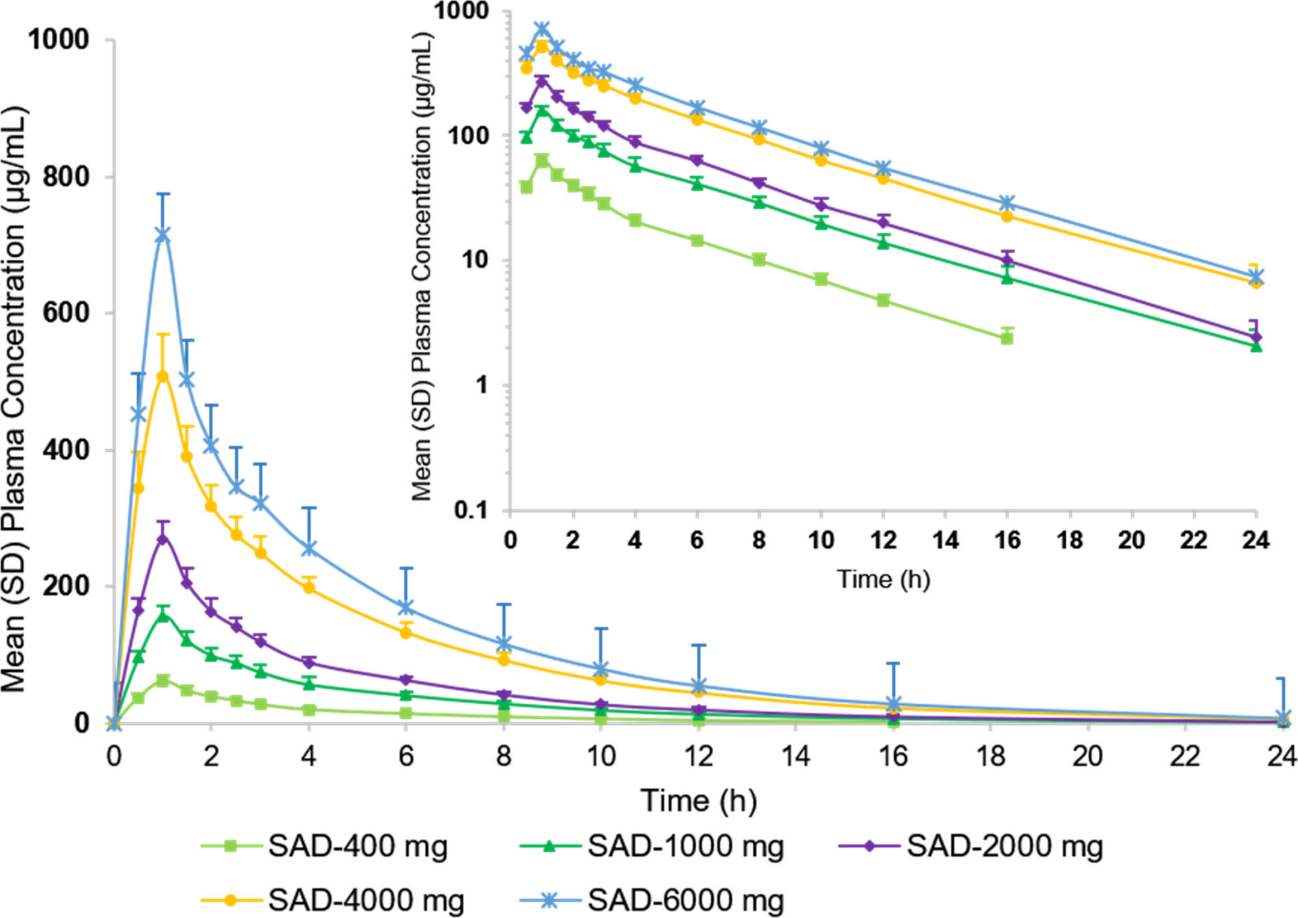
Mean (SD) plasma concentrations of HRS-8427 following single-dose administration at 0 to 24 h after the start of the initial infusion.

The mean plasma concentration profiles of unchanged HRS-8427 after the infusion of single doses ranging from 400 to 6,000 mg are shown in Fig. 1. A summary of the values of the PK parameters following the single dose administration of HRS-8427 is shown in Table 5. Mean values ranged from 61.85 ± 8.09 to 715.75 ± 76.67 µg/mL for C_max_; 245.72 ± 24.2 to 2,960.15 ± 257.28 h*ug/mL for AUC_0–t_ and 257.54 ± 24.82 to 2,983.09 ± 253.38 h*ug/mL for AUC_0-∞_, suggesting low interindividual variability for plasma exposure in all dose groups. The mean plasma t_1/2_ of HRS-8427 was 3.89 to 4.28 h. Estimates of the slopes for the C_max_, AUC_0–t_, and AUC_0-∞_ of HRS-8427 were 0.90 (90% confidence interval [CI]: 0.86–0.93), 0.92 (90% CI: 0.88–0.95), and 0.90 (90% CI: 0.87–0.94), respectively. None of the 90% CI included 1, and they were slightly below 1. The result suggests the increase in exposure levels of HRS-8427, within the 400–6,000 mg dose range, which is slightly lower than the increase in dose, indicating a generally linear pharmacokinetic profile. The data showed no apparent dose dependency for t_1/2_, whereas CL, Fe, and CL_r_ were slightly higher at doses above 1,000 mg than those in 400–1,000 mg.

**TABLE 5:** PK parameters for HRS-8427 following single intravenous infusions of 400–6,000 mg^*a*^^,^^*b*^

PK parameter(unit)	400 mg (*N* = 8)	1,000 mg (*N* = 8)	2,000 mg (*N* = 7)	4,000 mg (*N* = 8)	6,000 mg (*N* = 8)
C_max_(μg/mL)	61.85 ± 8.09	156.38 ± 14.56	269 ± 27.59	506.5 ± 61.80	715.75 ± 76.67
Tmax(h)^*a*^	1.00 (1.00 ~ 1.00)	1.00 (1.00 ~ 1.00)	1.00 (1.00 ~ 1.00)	1.00 (1.00 ~ 1.00)	1.00 (1.00 ~ 1.03)
AUC_0-t_ (h*μg/mL)	245.72 ± 24.20	690.12 ± 85.26	1,088.67 ± 79.7	2,289.88 ± 221.14	2,960.15 ± 257.28
AUC_0-∞_ (h*μg/mL)	257.54 ± 24.82	703.2 ± 88.54	1,102.85 ± 80.77	2,316.44 ± 219.4	2,983.09 ± 253.38
t1/2 (h)	3.94 ± 0.45	4.28 ± 0.53	3.89 ± 0.45	4.28 ± 0.54	4.12 ± 0.20
V_z_ (L)	8.89 ± 1.11	8.88 ± 1.35	10.23 ± 1.32	10.7 ± 1.27	12.03 ± 1.07
CL (L/h)	1.57 ± 0.16	1.44 ± 0.18	1.82 ± 0.15	1.74 ± 0.17	2.02 ± 0.17
Ae (mg)	248.50 ± 25.37	634.00 ± 66.51	1,421.85 ± 117.42	2,805.25 ± 196.13	4,267.22 ± 324.30
Fe (%)	62.13 ± 6.34	63.41 ± 6.65	71.10 ± 5.88	70.13 ± 4.91	71.12 ± 5.42
CLr (L/h)	0.97 ± 0.11	0.92 ± 0.17	1.30 ± 0.15	1.22 ± 0.15	1.39 ± 0.11

^*a*^ T_max_ are presented as median (min; max), and other parameters are presented as mean ± SD.

^*b*^ C_max_, peak concentrations; T_max_, time to reach peak concentrations; AUC_0-t_, area under the plasma concentration-time curve from 0 to the time of the last quantifiable concentration; AUC_0-∞_, area under the plasma concentration-time curve from time 0 to infinity; t_1/2_, elimination half-life; CL, total clearance; V_Z_, volume of distribution; Ae, Cumulative amount of drug excreted unchanged in urine; Fe, fraction of dose excreted unchanged into urine; and CLr, renal clearance of drug.

**Fig 2:**
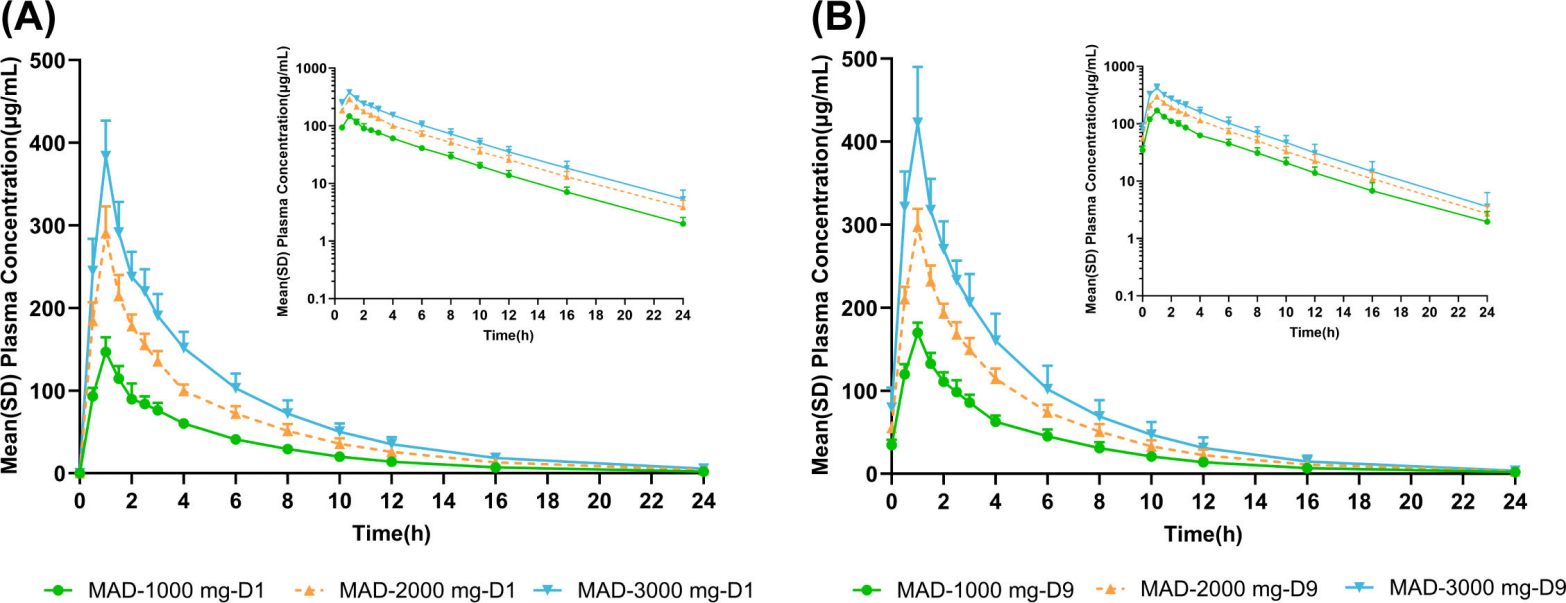
Mean (SD) plasma concentrations of HRS-8427 following multi-dose administration at 0–24 h after the start of the initial infusion on Days 1 (**A**) and 9 (**B**).

Figure 2 shows the mean plasma concentration profiles of HRS-84727 following multiple infusions in the 1,000 mg, 2,000 mg, and 3,000 mg groups. Plasma concentrations were similar after the first dosing (Day 1) and the last dose (Day 9), of which the accumulation ratios of C_max_ and AUC_0-_t were 1.09 to 1.16 and 1.03 to 1.16, respectively, indicating that there is little accumulation after multiple dosing. Notably, the mean plasma t_1/2_ of HRS-8427 was 4.24–4.26 h and 3.69–3.90 h on days 1 and 9, respectively, which were similar after multi-dose. A summary of the values of the PK parameters for HRS-8427 after multiple-dose administration is shown in Table 6.

**TABLE 6:** PK parameters for HRS-8427 following multi-intravenous infusions of 1,000 to 3,000 mg*^a^*^,^*^b^*

PK parameter(unit)	1,000 mg (*N* = 7)		2,000 mg (*N* = 8)		3,000 mg (*N* = 6)	
	Day 1	Day 9	Day 1	Day 9	Day 1	Day 9
C_max_(ug/mL)	146.86 ± 17.53	169.86 ± 12.06	290.5 ± 32.21	297.75 ± 21.41	383.67 ± 43.16	422.67 ± 67.40
T_max_(h)^*a*^	1.00 (1.00–1.00)	1.00 (1.00–1.05)	1.00 (1.00–1.02)	1.03 (1.00–1.07)	1.00 (0.98–1.10)	1.00 (1.00–1.05)
AUC_0-t_ (h*ug/mL)	682.86 ± 82.51	761.95 ± 105.62	1,249.08 ± 122.96	1,304.66 ± 127.58	1,741.82 ± 253.41	1,833.96 ± 391.4
AUC_0-8_ (h*ug/mL)	522.37 ± 57.00	603.02 ± 62.70	956.72 ± 80.83	1,044.82 ± 69.07	1,334 ± 174.10	1,466.13 ± 241.64
AUC_0-∞_ (h*ug/mL)	695.25 ± 85.82	774.46 ± 110.91	1,273.32 ± 130.41	1,320.05 ± 132.98	1,775.96 ± 265.81	1,846.68 ± 391.70
t_1/2_(h)	4.26 ± 0.22	3.88 ± 0.61	4.26 ± 0.35	3.9 ± 0.25	4.24 ± 0.58	3.69 ± 0.61
CL (L/h)	1.46 ± 0.18	1.68 ± 0.17	1.59 ± 0.17	1.92 ± 0.13	1.72 ± 0.27	2.09 ± 0.32
R_ac,AUC_	NA	1.16 ± 0.08	NA	1.09 ± 0.05	NA	1.11 ± 0.16
R_ac,Cmax_	NA	1.16 ± 0.09	NA	1.03 ± 0.10	NA	1.11 ± 0.17

^*a*^ T_max_ are presented as median (min; max), and other parameters are presented as mean ± SD.

^*b*^ C_max_, peak concentrations; T_max_, time to reach peak concentrations; AUC_0-t_, area under the plasma concentration-time curve from 0 to the time of the last quantifiable concentration; AUC_0-t_, area under the plasma concentration-time curve from 0 to the time of the last quantifiable concentration; AUC_0-8_, area under the concentration–time curve from 0 to 8 h; AUC_0-∞_, area under the plasma concentration-time curve from time 0 to infinity; t_1/2_, elimination half-life; CL, total body clearance; R_ac_, accumulation ratio, NA, not available.

Moreover, estimates of the slopes for the *C*_max_, AUC_0–t,ss_, and AUC_0-∞,ss_ of HRS-8427 were 0.90 (90% CI: 0.86–0.93), 0.92 (90% CI: 0.88–0.95), and 0.90 (90% CI: 0.87–0.94), respectively. None of the 90% CI contained 1, and they were slightly less than 1, which is consistent with the results from the SAD study.

### Urinary excretion

**Fig 3:**
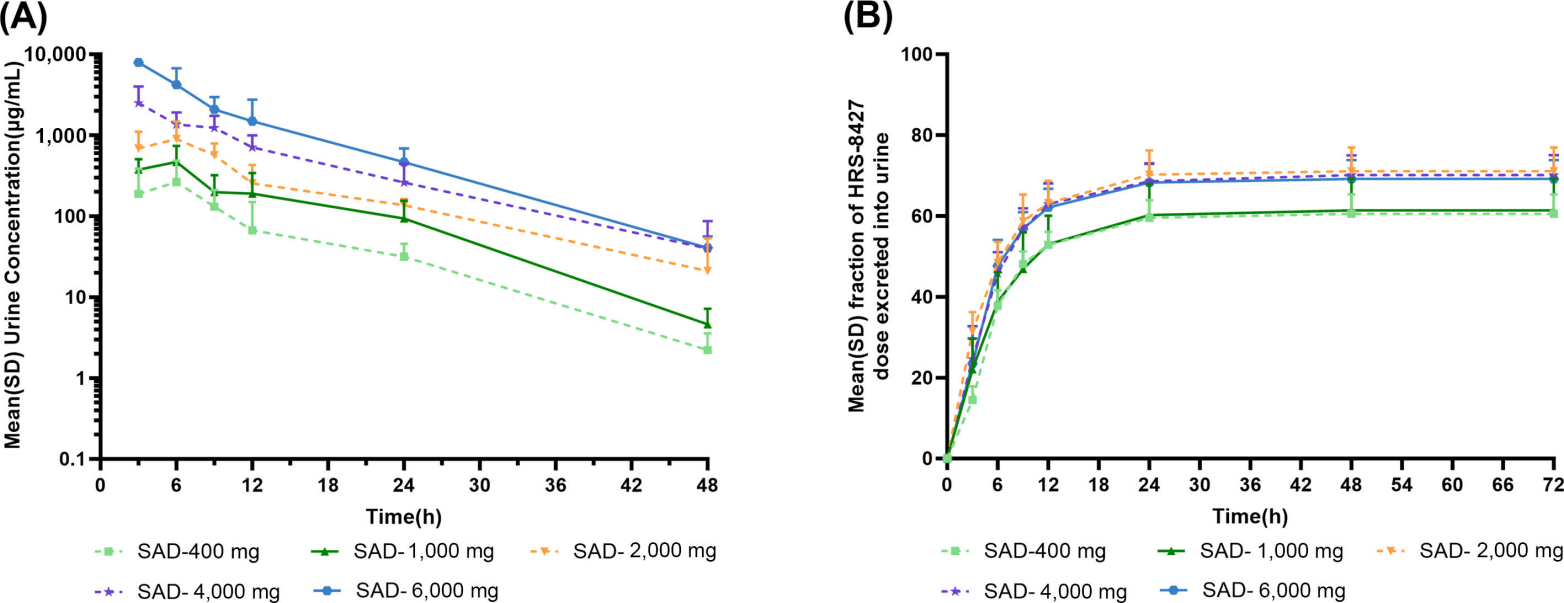
(**A**) Mean (SD) urine concentrations of HRS-8427 following single-dose administration. (**B**) Mean (SD) fraction of HRS-8427 dose excreted in urine following single-dose administration.

The mean urine concentration profiles of unchanged HRS-8427 after the infusion of single doses ranging from 400 to 6,000 mg are shown in Fig. 3. Dose-dependent increases in the urine concentrations appeared across the dose range of 400 to 6,000 mg. The mean urinary excretion ratio relative to the dose of HRS-8427 ranged from 62.13% to 71.10% unchanged drug (Fig. 3). Ranging from 400 mg to 6,000 mg, the majority of HRS-8427 excreted in urine within 24 h after dosing, with the mean HRS-8427 concentrations in urine within 24 h being above 10 µg/mL, and the amount of the drug in urine between 24 and 72 his minimal.

## DISCUSSION

HRS-8427 is a novel parenteral siderophore cephalosporin with *in vitro* and *in vivo* efficacy against gram-negative bacteria, including carbapenem-resistant *Enterobacteriaceae*, *P. aeruginosa*, and *A. baumannii*, which have shown that carbapenemase production is the most common resistance mechanism among clinical isolates in China, the United States, and the Middle East (3, 7).

In this study, we evaluated the safety, tolerability, and PK of HRS-8427 after single- and multiple-dose administrations in healthy Chinese subjects to support the clinical development of HRS-8427. The PK analysis showed approximately dose-proportional increases in exposure at doses ranging from 400 to 6,000 mg, with little accumulation in the multiple dosing study. The t_1/2_ was 3.89–4.28 h in Chinese subjects, which is longer than other cephalosporins, such as cefiderocol, ceftazidime, and cefepime (8–10). With the knowledge that cephalosporins demonstrate a time-dependent pharmacodynamic profile, a longer elimination half-life may provide more effective antibiotic exposure for successful therapy. Renal clearance, total clearance, and volume of distribution were modestly higher at doses above 1,000 mg compared with those at 400 mg and 1,000 mg, whereas elimination half-life remained unchanged. This increase is not likely to be attributable to changes in plasma protein binding, as *in vitro* studies demonstrated consistent protein binding across a concentration range of 10–500 µg/mL. The observed dose-dependent increase in clearance is more likely due to the saturation of tubular reabsorption at higher doses, leading to reduced reabsorption and enhanced renal elimination. The concomitant increase in the volume of distribution may reflect saturation of tissue binding. Together, the proportional increases in clearance and volume of distribution account for the stable half-life across doses. The observed accumulation ratio (1.1–1.2) was slightly lower than predicted for repeated dosing every 8 h with a 4 h half-life (~1.3) under linear PK, which is consistent with dose-dependent clearance due to saturated reabsorption reducing systemic exposure over time. However, given the limited sample size and the small magnitude of the observed differences, this finding is unlikely to be clinically meaningful, and additional data from Phase II and ongoing Phase III trials will be incorporated to further characterize the PK profile and elucidate the underlying mechanisms.

Similar to previous cephalosporin antibiotics (11), with 60%–70% of the administered dose of HRS-8427 being excreted in the urine as unchanged parent drug, further study, such as a mass-balance trial, is ongoing to characterize the metabolism and excretion of HRS8427. Moreover, a comprehensive understanding of the pharmacokinetics of HRS-8427 in patients with varying degrees of renal impairment is necessary to provide rational schedules for treatment of serious infections in patients with compromised renal function, as renal impairment represents a prevalent comorbidity among patients with gram-negative bacterial infections.

Our study demonstrated that HRS-8427 was well tolerated at doses of up to 3,000 mg q8h in healthy volunteers. No serious or severe AEs were reported in either study. In the SAD study, a total of nine TRAEs were reported in the treatment group, with an incidence rate of 20.0% (8/40). The TRAEs occurring in ≥2 subjects were proteinuria (one case in the 1,000 mg group and one case in the 6,000 mg group). The incidence rate of TRAEs in the placebo group was 10.0% (1/10). All TRAEs were of mild severity, and no significant trends or differences were observed between groups, with no apparent dose dependency for HRS-8427. In the MAD study, a total of 34 TRAEs were reported in the treatment group, with an incidence rate of 60.9% (14/23). The incidence rate of TRAEs in the placebo group was 16.7% (1/6). All TRAEs were of mild severity. The occurrence of proteinuria present increased with dose, and all cases resolved or recovered within 1 week after reporting. No significant trends or dose dependency were observed for other TRAEs across the groups. Hypersensitivity reactions, nausea, and headaches were only observed in the 3,000 mg dose group, suggesting that HRS-8427 at the 3,000 mg multiple-dose regimen may increase the risk of hypersensitivity, gastrointestinal disorders, and neurological disorders. In alignment with other cephalosporins, hypersensitivity is a safety concern in clinical practice (12). Skin testing for cephalosporins has not been well-validated (13, 14), as each subject underwent a skin test before HRS-8427 administration, but this does not seem to be a good way to avoid hypersensitivity.

This study was conducted in healthy Chinese subjects, who have a lower average body weight compared with populations in Western regions such as the United States. Therefore, caution should be exercised when extrapolating the findings to broader ethnic or geographic populations. However, the enrolled subjects are representative of the target Chinese patient population in terms of body weight and demographic characteristics, supporting the relevance of the data for further clinical development in this group.

In conclusion, this study indicates that single doses up to 6,000 mg and multiple intravenous doses up to 3,000 mg of HRS-8427 are well tolerated in healthy subjects, and generally, dose proportionality for the PK parameters was observed. The study findings establish a critical translational framework for advancing HRS-8427 through subsequent clinical development of targeting multidrug-resistant gram-negative bacterial infections. Based on PK data in this study from healthy volunteers and renal-impaired patients, a population PK and PK/PD model has been developed, and the probability of target attainment (PTA) analyses was used to select a dose level in the Phase II study in patients with complicated urinary tract infections (unpublished). Dose regimen of 2 g q8h has been demonstrated efficacious and safe in this Phase II study and is being evaluated in the ongoing Phase III trial in patients with cUTI/AP and ongoing Phase II trial in patients with bacterial pneumonia.

## MATERIALS AND METHODS

### Subjects

Eligible subjects were healthy males or females aged 18–45 years with a weight ≥50.0 kg for males and ≥45.0 kg for females and a body mass index of 19.0–26.0 kg/m^2^. Subjects with, but not limited to, central nerve/psychiatric disorders and respiratory, cardiovascular, gastrointestinal, hematological, and endocrine disorders, which were considered clinically significant in the study, were excluded. Subjects were also excluded if they had clinically significant abnormalities on physical examination, vital signs, 12-lead electrocardiogram (ECG), or laboratory tests (including hematology, chemistry, urinalysis, and pregnancy tests). Subjects with a history of allergy to any drugs, including beta-lactam antibiotics, were excluded. All subjects provided written informed consent prior to the start of the study. The study was performed at the Second Hospital of Anhui Medical University, China, with the approval of the institutional ethics committee and in accordance with the principles of the Declaration of Helsinki and good clinical practice guidelines.

### Study design

This was a Phase I, single-center, randomized, double-blind, placebo-controlled study conducted in two parts: a single-ascending-dose study (SAD) and a multiple-ascending-dose study (MAD). This study was registered at the China Center for Drug Evaluation under the identifier number CTR20222003. All participants provided written informed consent prior to participation.

The single-dose study was planned to include up to five single doses of HRS-8427 in five cohorts (400, 1,000, 2,000, 4,000, and 6,000 mg). A total of 50 healthy Chinese males and females (eight individuals receiving the active drug and two individuals receiving a placebo per cohort) were planned for enrollment and participated in the single-dose study. The screening and eligibility assessments occurred between day 14 (14 days before day 1, defined as the day of study drug administration) and day 1. Eligible subjects were admitted to the study center on day 1, confined to the study center until day 4, and returned to the study center on day 7 for follow-up.

The multiple-dose study was planned to include up to three doses of HRS-8427 (1,000 mg, 2,000 mg, and 3,000 mg) in three groups. A total of 30 healthy Chinese males and females (eight individuals receiving the active drug and two individuals receiving a placebo per cohort) were planned for enrollment and participated in the multiple-dose study. Subjects received 1 h IV infusions of HRS8427 or saline placebo on days 2–8 every 8 h (q8h). On days 1 and 9, subjects received only a single dose of their assigned treatment. The screening and eligibility assessments occurred between day 14 (14 days before day 1, defined as the day of study drug administration) and day 1. Eligible subjects were admitted to the study center on day 1, confined to the study center until day 11, and returned to the study center on day 16 for follow-up.

### Safety evaluation

Safety was assessed through assessment of TEAEs, physical examinations, assessment of vital signs, 12-lead and continuous electrocardiogram (ECG) recordings, and performance of laboratory tests (hematology, blood chemistry, including iron and total iron-binding capacity, and urinalysis). All TEAEs were evaluated by the investigator for severity and relationship to the study drug. All AEs were coded using the Medical Dictionary for Regulatory Activities (MedDRA), version 25.

### Sample collection and analysis

For the SAD, serial blood samples were collected prior to infusion (0 h) and at 0.5 (during infusion), 1.0 (just before completion of infusion), 1.5, 2.0, 2.5, 3.0, 4.0, 6.0, 8.0, 10, 12, 16, 24, 36, and 48 h after the start of infusion. Urine samples were collected prior to infusion (−12h to 0 h) and 0–3, 3–6, 6–9, 9–12, 12–24, 24–48, and 48–72 h from the start of the infusion.

For the multiple-dose study, serial blood samples were collected as follows: at 0, 0.5, 1.0, 1.5, 2.0, 2.5, 3.0, 4.0, 6.0, 8.0, 10, 12, 16, and 24 h from the start of the first (morning) infusion on day 1; prior to the morning infusion (0 h) on days 6, 7, and 8; and at 0, 0.5, 1.0, 1.5, 2.0, 2.5, 3.0, 4.0, 6.0, 8.0, 10, 12, 16, 24, 36, and 48 h from the start of the final (morning) infusion on day 9.

Blood samples were collected into pre-chilled (2°C–8°C, ≥30 min) K₂EDTA vacuum tubes. At each time point, 4 mL of blood was collected and processed within 30 min: centrifuged at 1,700 × *g* for 10 min at 2°C–8°C, followed by plasma separation into cryovials. The resultant plasma was stored frozen at −60°C to −90°C until analysis. Urine samples were stored at 2°C–8°C immediately after collection. Upon completion of each collection interval, total urine volume was determined by weight, and 1 mL aliquots were transferred to sample tubes within 20 min for analysis. Urine samples were stored frozen at −60°C or below until analysis. All sample handling procedures were performed under yellow light conditions.

The concentrations of HRS-8427 in human plasma and urine were determined by a liquid chromatography (LC)-tandem mass spectrometry (MS/MS) method. The lower limit of quantification of HRS-8427 was 1.0 µg/mL in plasma and 1.0 µg/mL in urine. The assay was linear from 1.0 to 600 µg/mL and 1.0 to 1,000 µg/mL for plasma and urine, respectively. The intra- and inter-batch precision of the assay was <9.5% and <6.1% for plasma and urine, respectively. The accuracy of the assay was –2.4% to 1.5% and 1.1% to 3.0% for plasma and urine, respectively.

### Pharmacokinetic and statistical analyses

All statistical analyses were conducted using SAS version 9.4 (SAS Institute Inc., Cary, NC, USA). PK parameters were determined by noncompartmental analysis using Phoenix WinNonlin V8.3 (Certara Inc., Princeton, NJ, USA).

The following plasma and urine PK parameters were evaluated: maximum plasma concentration (C_max_), time to C_max_ (t_max_), area under the plasma concentration-time curve from 0 to the time of the last quantifiable concentration (AUC _(0-t)_), area under the plasma concentration-time curve from time 0 to infinity (AUC_0-∞_), terminal half-life (t_1/2_), total body clearance (CL), and volume of distribution (V_z_). AUC was calculated with the linear-up/log-down trapezoidal method for the extrapolation. Cumulative amount of drug excreted unchanged in urine (Ae), fraction of dose excreted unchanged into urine (Fe), and renal clearance of drug (CL_r_) were estimated for each subject with urinary excretion data.

The dose proportionality of the PK parameters was examined by using a power model. The model structure was defined as: Ln (PK parameter) = Intercept + β*Ln (Dose) + Error), where β is the slope of the linear model used to measure the linear proportionality between the exposure and dose. A descriptive point estimate and a 90% CI for the slope β are obtained based on the linear regression model. If the 90% CI for β includes 1, it suggests a linear proportionality between the exposure and dose.
